# The learning curve of TaTME for mid-low rectal cancer: a comprehensive analysis from a five-year institutional experience

**DOI:** 10.1007/s00464-020-08115-0

**Published:** 2020-10-26

**Authors:** Roberto Persiani, Annamaria Agnes, Francesco Belia, Domenico D’Ugo, Alberto Biondi

**Affiliations:** 1 Dipartimento Scienze Mediche e Chirurgiche, UOC di Chirurgia Generale, Fondazione Policlinico Universitario A. Gemelli IRCCS, Largo Agostino Gemelli n. 8, 00168 Rome, Italy; 2 Università Cattolica del Sacro Cuore, Largo Francesco Vito n. 1, 00168 Rome, Italy

**Keywords:** TaTME, Transanal total mesorectal excision, Learning curve, CUSUM, Recurrence

## Abstract

**Background:**

Transanal total mesorectal excision (TaTME) was introduced in 2009 as a dedicated approach for the treatment of mid-low rectal cancer. We aimed to describe and discuss the learning curve for 121 consecutive TaTME procedures performed by the same team.

**Methods:**

The primary outcome was the number of operations required to decrease the mean operative time (mOT). The secondary outcomes were the number of operations required to decrease the major complication (MC) rate, the anastomotic leakage (AL) rate, the clinical anastomotic failure rate, and the reoperation rate. A cumulative sum (CUSUM) curve analysis was used to identify the inflection points. As an integrative analysis, Bernoulli CUSUM curves, risk-adjusted CUSUM curves based on the observed-expected outcomes, and CUSUM curves targeting results reported in the literature were created.

**Results:**

Seventy-one cases were needed to overcome the OT learning curve sufficiently to reach mastery. The MC and reoperation rates started to decrease after the 54th case and further decreased after the 69th case. The AL rate started to decrease after the 27th case and remained stable at 5–5.1%. The comparison between the different phases of the learning curves confirmed these turning points.

**Conclusions:**

TaTME had a learning curve of 71 cases for the mOT, 55–69 cases for MCs and reoperation, and 27 cases for AL. According to our results, attention should be paid during the first part of the learning curve to avoid an increased rate of MCs and AL.

**Electronic supplementary material:**

The online version of this article (10.1007/s00464-020-08115-0) contains supplementary material, which is available to authorized users.

Rectal resection with total mesorectal excision (TME) is accepted as the surgical standard for the treatment of rectal cancer [1, 2]. The non-inferiority of laparoscopic compared to open TME in terms of disease-free survival (DFS) has been demonstrated in the phase III COREAN and COLOR II trials [3, 4]. However, two recent randomized controlled trials (RCTs) failed to demonstrate the non-inferiority of the minimally invasive approach compared to the open approach in terms of pathological results [5, 6]. Laparoscopic TME is associated with increased surgical difficulty when some clinical and anatomical factors, such as a high BMI, a narrow pelvis, or bulky or very low tumors, are present. For this reason, transanal total mesorectal excision (TaTME) has been proposed as a dedicated approach for the treatment of mid and low rectal cancers [7]. Since its introduction in 2009, there has been a lively debate around TaTME. In centers where TaTME is routinely performed, short- and long-term results have been reported as non-inferior to those of laparoscopic TME, with advantages in terms of short-term outcomes, such as postoperative complications and readmission, and non-inferiority in terms of long-term oncological outcomes [8]. However, there have been some reports of specific surgical complications associated with TaTME (urethral injuries, pelvic wall injuries, carbon dioxide embolism, nerve damage) [9, 10]. A more worrisome issue is the unexpectedly high rate of early locoregional recurrence reported in two studies analyzing oncological outcomes in the early phase of TaTME implementation [10, 11]. In light of these reports, the need for standardized surgical education and proper training in this technique have been advocated [12]. Theoretically, patients’ clinical outcomes are supposed to be related to surgeons’ experience with a specific procedure, and the definition of a learning curve is imperative to define the number of procedures to be strictly monitored and supervised before reaching proficiency. To date, only three observational studies have reported a learning curve analysis in TaTME [13–15].

Analyzing different outcomes, these papers identified a cutoff point of 40 cases for improved postoperative short-term results [13], 45–51 cases for high-quality pathological specimens [14], and 50 cases for reducing the anastomotic leakage (AL) rate [15]. However, due to some heterogeneity in the case series and the small number of studies in the literature, the learning curve for TaTME is not yet standardized.

At Fondazione Policlinico Universitario A. Gemelli IRCSS (Rome, Italy), TaTME has been routinely used to treat mid-low rectal cancer since 2015 [16]. The aim of this study was to evaluate the learning curve for TaTME in this series.

## Methods

### Patient selection

One hundred twenty-eight patients underwent elective TaTME for rectal cancer between 30 April 2015 and 31 December 2019 at Fondazione Policlinico Universitario A. Gemelli IRCCS, Rome. All patient data were included in a prospectively recorded institutional database and retrospectively analyzed for the purposes of this study. Patients considered eligible for this study were adults with mid-low rectal neoplasms undergoing elective TaTME. Patients undergoing transanal rectal resection for inflammatory bowel disease (*n* = 5) and patients undergoing TaTME in reoperation for locoregional recurrence after previous rectal resection (*n* = 2) were excluded from the study cohort. The Institutional Review Board/Ethical committee approved this study (approval number is 2760906—Current Research Project registered in the Italian Research Workflow).

### Data collection

Data were retrospectively collected from the institutional database. The collected data included sex, age, BMI, previous abdominal surgery, Charlson comorbidity index (CCI), ASA score, preoperative tumor stage, preoperative neoadjuvant treatment, preoperative hemoglobin, preoperative albumin, distance of the tumor from the anorectal junction (measured by magnetic resonance imaging (MRI)), operative time (OT), type of colorectal reconstruction, creation of diverting ileostomy, tumor dimension, p/yp stage, distance from the distal margin on pathological staging, circumferential resection margin (CRM) status, postoperative stay, 30-day postoperative complications (all postoperative complications, reoperation, readmission and mortality), presence of early and late AL, anastomotic stenosis, and follow-up data (overall survival (OS), local and distant DFS). The tumor stage was determined according to the 8th edition of the AJCC-TNM classification [17]. Thirty-day postoperative morbidities were classified according to the Clavien–Dindo classification [18]; major postoperative complications were defined as complications of grade 3 to 5. Thirty-day postoperative readmission was considered a postoperative complication scored according to the Clavien–Dindo classification. Postoperative leakage was defined according to the definition provided by the International Study Group of Rectal Cancer [19]. Clinical anastomotic failure was defined as clinically relevant AL or stenosis requiring re-anastomosis or a definitive stoma, while anastomotic failure was defined according to the definition of Penna et al. [9]. A positive CRM was defined as a CRM < 1 mm. The local recurrence rate was calculated in patients with a minimum follow-up of 24 months.

### Surgical technique and perioperative management

The abdominal procedure was performed as a laparoscopic rectal resection procedure, namely, a 3/4 trocar technique that included central vascular ligation, mobilization of the splenic flexure, medial to lateral mesocolic mobilization and incision of the pelvic peritoneal reflection. The transanal procedure was performed as previously described [16]. All surgical procedures were performed by the same surgical team. The first eight procedures were performed using a single-team approach, while from the ninth case on, a synchronous double-team approach was adopted. All patients followed a standardized perioperative enhanced recovery after surgery (ERAS) protocol [20].

### Oncological management

Initial tumor staging was performed using thoracoabdominal computed tomography (CT) and pelvic MRI. Patients with locally advanced rectal cancer (cT3-T4, nodal involvement) were treated with neoadjuvant chemoradiotherapy. After neoadjuvant therapy, patients were locally restaged by MRI. Surgery was performed at a minimum of 8 weeks after the end of radiotherapy. After surgery, according to the pathological staging, patients received a postoperative evaluation with a contingent indication for the administration of postoperative chemotherapy.

### Study outcomes

The primary outcome of this study was the number of operations required to decrease the mean operative time (mOT). The secondary outcomes were the number of operations required to decrease the postoperative stay, the postoperative complication rate, the major complication (MC) rate, the AL rate, the anastomotic failure rate, the reoperation rate, and the readmission rate, with characterization of the different learning curves and comparison of the learning curves for every outcome.

### Statistical analysis

The learning curve analysis was performed according to the cumulative sum CUSUM method to explore the relationship between the primary outcomes and the sequence number of the TaTME procedure [21]. The simple CUSUM series was defined for every case as CUSUM_n_ = ∑(*X*_n_ – *X*_0_) + CUSUM_n-1_, where *X*_n_ represents the individual measurement, and *X*_0_ is a predetermined reference level. *X*_0_ was set as the mean for all cases in the first analysis. The CUSUM score was plotted against the sequence of operations. The inflection point of the learning curve was defined as the point where the curve started to descend gradually and was considered the end of the learning curve [22].

As a complement to the CUSUM series, a two-sided Bernoulli CUSUM chart was plotted to detect consistency with the mean or “alarm signals” in the surgical performance [23]. The point at which the OT became consistent with the mean, without further significant changes in terms of the mOT, was defined as the point of “mastery” of the technique [22]. The control limits for the “alarm signals” were set at ± 4*σD (*σD* = standard deviation). The Bernoulli CUSUM_max_ and CUSUM_min_ were defined as CUSUM_maxn_ = max (0, *X*_n_ – i_0 _+ *σD*/2 + CUSUM_maxn-1_), where the max (0,) yields 0 when a negative value is calculated, and CUSUM_minn_ = min (0, *X*_n_ – *X*_0—_*σD*/2 + CUSUM_maxn-1_), where the min (0,) yields 0 when a positive value is calculated.

Then, to account for possible patient-related confounders, a risk-adjusted CUSUM (RA-CUSUM) analysis based on observed-expected (O–E) statistics was performed [24]. A set of multivariate regression analyses was conducted for every outcome. For linear regressions (dependent variables: OT, postoperative stay), a backward selection model was applied, while for logistic regressions, a backward (dependent variable: postoperative complications) or forward (dependent variables: MCs, AL, anastomotic failure, reoperation, readmission) selection model was applied, according to the number of events *per* outcome. The risk scores for the expected outcomes were calculated according to the constant and the regression coefficients of the variables in the final model. For the logistic regression equation, a conversion from odds ratios (ORs) to risk probability coefficients was performed according to the following formula *p* = exp(OR)/[1 + exp(OR)]. The risk scores derived from the regression equations were substituted for *X*_0_ for every single case in the CUSUM equation. A two-sided Bernoulli CUSUM chart was plotted for these results using the σD of the observed outcome [25].

Last, a second simple CUSUM analysis where X_0_ was set as the specific mean reported in the literature was performed. The paper by Penna et al. [9] reporting data from the International TaTME Registry was used as a reference for the positive distal margin (0.7%)/positive CRM rate (4.1%), the postoperative complication rate (35.2%), the MC rate (13.2%), the AL rate (9.8%), and the reoperation rate (8%).

Patients were then divided into early-experience and late-experience groups according to the cutoff points of the CUSUM graphs. Quantitative data are reported as either the mean ± standard deviation (range) or median ± range. Continuous variables were analyzed using Student’s t test or ANOVA. Qualitative data are reported as the number of patients (percentage of patients) and were compared with Pearson *χ*^2^ or Fisher’s exact test.

All analyses were performed using Microsoft Office Excel and IBM SPSS, version 23 (IBM Co., Armonk, NY, USA). All tests were 2-sided with a significance level set at 0.05.

## Results

**Fig. 1:**
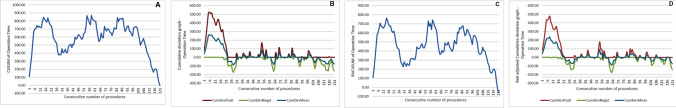
CUSUM curves associated with operative time. **A** Simple CUSUM curve, **B** Bernoulli Cumulative Deviation Curves for simple CUSUM, **C** RA-CUSUM curves, **and D** Bernoulli Cumulative Deviation Curves for RA-CUSUM. *CumDevPosit* Cumulative sum of the positive deviations, *CumDevNeg* Cumulative sum of the negative deviations, *CumDevMean* mean of the CumDevPos and CumDevNeg values

**Fig. 2:**
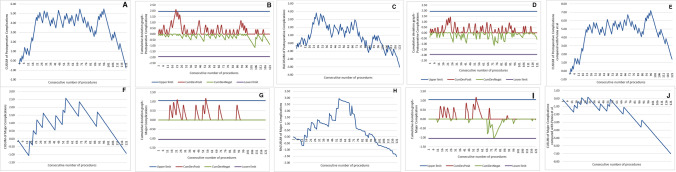
CUSUM curves associated with postoperative complications and major complications. **A**–**F** simple CUSUM curve, **B**–**G** Bernoulli Cumulative Deviation Curves for simple CUSUM, **C**–**H** RA-CUSUM curves, **D**–**I** Bernoulli Cumulative Deviation Curves for RA-CUSUM, and **E**–**J** simple CUSUM curve using a reference mean from the literature. *CumDevPosit* cumulative sum of the positive deviations, *CumDevNeg* cumulative sum of the negative deviations, *CumDevMean* mean of the CumDevPos and CumDevNeg values

**Fig. 3:**
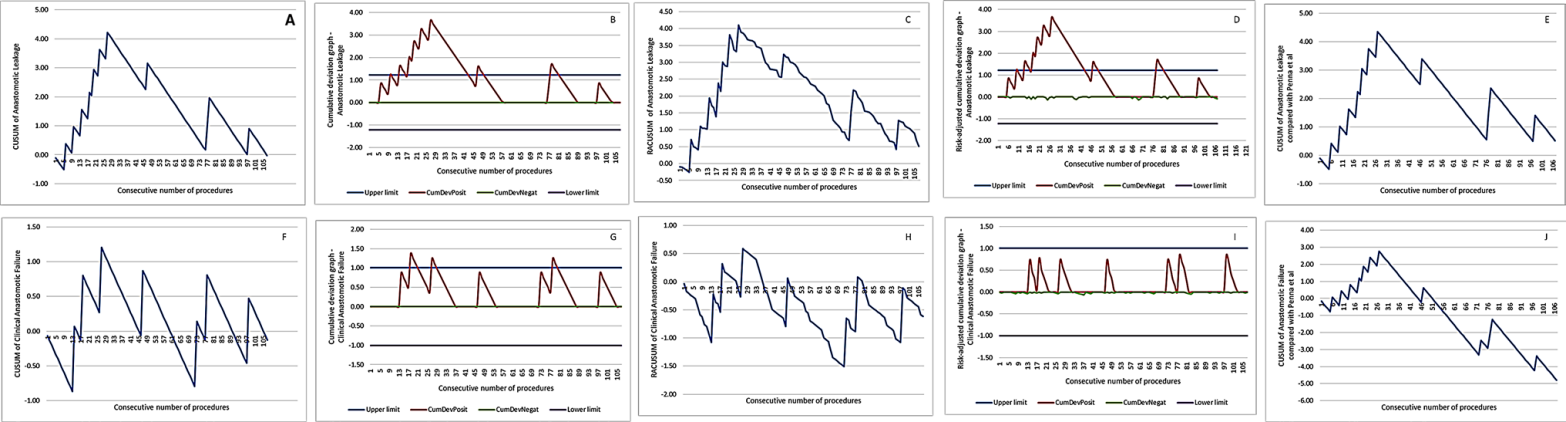
CUSUM curves associated with anastomotic leak, clinical anastomotic failure, and anastomotic failure. **A**–**F** simple CUSUM curve, **B**–**G** Bernoulli Cumulative Deviation Curves for simple CUSUM, **C**–**H** RA-CUSUM curves, **D**–**I** Bernoulli Cumulative Deviation Curves for RA-CUSUM, and** E**–**J** simple CUSUM curve using a reference mean from the literature. *CumDevPosit* cumulative sum of the positive deviations, *CumDevNeg* cumulative sum of the negative deviations, *CumDevMean* mean of the CumDevPos and CumDevNeg values

In this study, 121 consecutive patients were selected for the learning curve analysis according to the inclusion and exclusion criteria. The clinicopathological characteristics of the entire cohort are reported in Table 1. The equations obtained from the linear and logistic regression analyses used for the RA-CUSUM O-E analysis are presented in Table 2. No RA-CUSUM analysis was performed for the positive CRM rate due to the small number of positive cases (*n* = 3). The CUSUM curves are presented in Figs. 1, 2, and 3.

**Table 1:** Clinicopathologic, operative, and short-term outcomes of 121 patients undergoing TaTME

Variable	Values
Age, mean ± SD (range)	70 ± 11 (36–94)
Sex	
Male	80 (66.1%)
Female	41 (33.9%)
BMI, kg/m^2^, mean ± SD (range)	25.2 ± 3.9 (14–41)
Charlson Comorbidity Index, mean ± SD (range)	6 ± 3 (0–19)
ASA ≥ 3	
Yes	17 (14.1%)
No	101 (83.5%)
Missing	3 (2.5%)
Hemoglobin, g/dl, mean ± SD (range)	12.8 ± 1.8 (8.9–17.6)
Albumin, mg/dl, mean ± SD (range)	39.7 ± 4.5 (26–62)
Active smoker	
Yes	18 (14.9%)
No	101 (83.5%)
Missing	2 (1.7%)
Previous laparotomy	
Yes	53 (43.8%)
No	67 (55.4%)
Missing	1 (0.8%)
Height of the tumor from anorectal junction determined at the MRI	
≤ 50 mm	57 (47.1%)
> 50 mm ≤ 100 mm	61 (50.4%)
> 100 mm	3 (2,5%)
Height of the tumor from anorectal junction determined at the MRI, mm, median (range)	60 (15–120)
cStage	
0	6 (5%)
I	13 (10.7%)
II	15 (12.4%)
III	82 (67.8%)
IV	5 (4.1%)
Neoadjuvant chemoradiation	
Yes	79 (65.3%)
No	42 (34.7%)
Operative time, min, mean ± SD (range)	284 ± 58 (180–440)
Combined approach	
Yes (two teams)	113 (93.4%)
No (one team)	8 (6.6%)
Reconstruction (colorectal anastomosis)	
Yes	108 (89.3%)
No	13 (10.7%)
Protective ostomy*	
Yes	101 (93.5%)
No	7 (6.5%)
Conversion	
Yes	1 (0.8%)
No	120 (99.2%)
Tumor max size, mm, mean ± SD (range)	28 ± 19 (0–150)
Distal margin, mm, mean ± SD (range)	19 ± 11 (2–50)
CRM involvement	
Yes	4 (3.3%)
No	117 (96.7%)
Quality of specimen (Quirke)	Complete 107 (88.4%) Nearly complete 11 (9.1%) Incomplete 3 (2.5%)
Evaluated lymph nodes, mean ± SD (range)	12 ± 5 (0–26)
p/yp Stage	
Complete response/Stage 0	26 (21.5%)
I	37 (30.6%)
II	27 (22.3%)
III	27 (22.3%)
IV	4 (3.3%)
Postoperative hospital stay, days, mean ± SD (range)	6 ± 3 (3–19)
Postoperative complications	
Yes	44 (36.4%)
No	77 (63.6%)
Clavien–Dindo grade	
0	77 (63.6%)
I	21 (17.4%)
II	14 (11.6%)
III	6 (5%)
IV	1 (0.8%)
V	2 (1.7%)
Anastomotic leakage*	
Yes	11 (10.2%)
No	96 (88.9%)
Missing	1 (0.9%)
Clinical anastomotic failure*	
Yes	7 (6.5%)
No	98 (90.7%)
Missing	3 (2.7%)
Anastomotic failure according to Penna et al.*	
Yes	12 (11.1%)
No	93 (86.1%)
Missing	3 (2.7%)
Reoperation	
Yes	7 (5.8%)
No	114 (94.2%)
Readmission	
Yes	15 (11.4%)
No	106 (88.6%)

*Calculated on 108 patients with CR anastomosis

**Table 2:** Equations for the calculation of the expected outcomes derived from the linear and logistic regression analyses

Operative time (min)	= 190.967 + (− 29.89 * Female Sex) + (2.522 * BMI) + (21.786 * Node Positivity) + (41.314 * Diverting Ileostomy)
Postoperative complications (OR)	= 2.723 + (− 1.362 * Neoadjuvant Therapy) + (0.194 * Charlson Index) + (0.007* Operative Time)
Major complications (OR)	= − 13.792 + (0.446 * Charlson Index) + (0.312 * BMI)
Reoperation (OR)	= − 11.507 + (0.314 * Charlson Index) + (0.249 * BMI)
Anastomotic leakage (OR)	= − 9.62 + (0.102 * Age)
Clinical anastomotic failure (OR)	= − 1.792 + (− 1.626 * Neoadjuvant Therapy)

### CUSUM charts for the surgical outcomes

#### OT (Fig. 1A–D)

The mOT in the cohort was 284 ± 54 min. The CUSUM analysis identified an initial phase of increase up to the 18th procedure, then a decrease down to the 30th procedure, followed by a second increase up to the 54th procedure. Then, there was a plateau phase until the 87th procedure, followed by a progressive decrease in the mOT. The RA-CUSUM O-E analysis identified a similar pattern with a peak up to the 54th case, a temporary decrease followed by a moderate increase, and then a consistent decrease in the mOT after the 87th procedure. The Bernoulli CUSUM charts detected a mOT above the upper control limit until the 19th procedure. “Mastery” of the procedure was reached at the 71st procedure.

#### Postoperative complications (Fig. 2A–E)

The rate of postoperative complications was 36.3%. The CUSUM analysis identified an initial phase of increasing postoperative complications up to the 24th procedure, followed by a plateau to the 69th procedure, a progressive decrease down to the 85th procedure, a second increase up to the 96th procedure, and then a decrease. The RA-CUSUM analysis identified an increase up to the 24th procedure, followed by a plateau to the 69th procedure, and then a progressive decrease. The Bernoulli CUSUM charts detected a postoperative complication rate above the upper control limit for the 21st procedure. Comparison with the values of the International TaTME Registry showed a long plateau phase from the 24th to the 108th procedure, with peaks at the 69th and 96th procedures.

#### MCs (Fig. 2F–J)

The MC rate was 7.4%. The CUSUM analysis identified a progressive increase in the curve up to the 54th procedure. A second lesser peak was detected at the 69th procedure. The RA-CUSUM analysis identified a peak at the 54th procedure, followed by a decrease. The Bernoulli CUSUM charts identified a MC rate that was above the upper control limit from the 53rd to the 55th procedure. Comparison with the values of the International TaTME Registry showed a turning point at the 54th procedure.

#### Reoperation (Supplementary Fig. 1sa–e)

The reoperation rate was 5.8% (7 patient). The reasons of the reoperations were as follows: in 1 patient a complete disruption of the anastomosis that required an abdominoperineal resection; in 2 patients ischemia of the anastomotic descending colon that required resection and terminal colostomy; in 2 patients a malfunction of the ileostomy that required revision; in 1 patient small bowel injury that required exploratory laparoscopy and suture; and in 1 patient early postoperative bowel obstruction due to a internal hernia that required exploratory laparotomy. The reoperation rate increased up to the 22nd procedure and then presented a plateau phase with a higher peak at the 69th procedure and a turning point at the 89th procedure. The RA-CUSUM analysis identified a progressive increase up to the 54th procedure and then a progressive decrease. The Bernoulli CUSUM charts identified a reoperation rate that was above the control limit from the 18th to the 31st procedure in the unadjusted graph and for the 22nd and 23rd procedures in the O-E statistic-based graph. Comparison with the values of the International TaTME Registry showed a peak at the 22nd procedure, a plateau and a then turning point at the 69th procedure.

#### AL (Fig. 3A–E)

The AL rate was 10.2%. The rate of AL increased up to the 27th case and then started to decrease, presenting a second peak up to the 47th procedure, followed by a progressive decrease. At the 78th procedure, there was another minor peak, followed by a decrease. The RA-CUSUM analysis was consistent with the previous analysis. The Bernoulli CUSUM charts detected an AL rate that remained above the control limit from the 16th to the 42nd procedure. The comparison with the values of the International TaTME Registry was consistent with the previous analyses.

#### Clinical anastomotic failure (Fig. 3F–J)

The rate of clinical anastomotic failure was 6.5%. The rate of clinical anastomotic failure increased up to the 27th case and then presented two minor peaks at the 47th and 78th cases. The RA-CUSUM analysis identified a peak at the 27th case and then a substantial decrease in the curve below the reference limit. The Bernoulli CUSUM chart detected an AL rate above the control limit from the 18th to the 29th procedure and from the 78th to the 80th procedure, while the O-E Bernoulli CUSUM chart described a curve below the control limit for all cases. Comparison of the anastomotic failure rate according to the definition of the International TaTME Registry [9] was 11.1% and showed a peak at the 27th procedure, followed by a progressive decrease.

### Comparisons between the clinical outcomes in the different learning phases

According to the visual analysis of the learning curves, cutoff points were defined at the turning point for the OT at the 54th and 87th procedures, for MCs and reoperation at the 54th and 69th procedures, and for AL at the 27th and 47th procedures. Comparison of the OT among the three phases (1–54, 54–87, 88–121) confirmed a significant difference and a progressive decrease in the OT (300 ± 59 vs 283 ± 58 vs 259 ± 46, *p* = 0.005). The rate of postoperative complications was significantly decreased from the first phase to the second phase using both the 54th and 69th procedure as turning points (*p* = 0.041 and *p* = 0.024, respectively). The rates of MCs, reoperation and readmission showed no significant differences, both using the 54th (*p* = 0.076, *p* = 0.240 and *p* = 0.067, respectively) and the 69th procedure (*p* = 0.076, *p* = 0.237, *p* = 0.055) as turning points. However, the crude rates of postoperative complications (46.3% vs. 40 vs. 25%), MCs (13% vs 6.7% vs 1.9%), reoperation (9.3% vs 6.7% vs 1.9%), and readmission (18.5% vs 13.3% vs 5.8%) all decreased from the early (1–54) to the middle (55–69) and late (70–121) phases, respectively.

Patients in the early (1–54) and middle-late (55–121) phases demonstrated a higher CCI (*p* = 0.040), a lower preoperative hemoglobin level (*p* = 0.009) and a higher rate of treatment with neoadjuvant radio-chemotherapy (*p* = 0.016) (Supplementary Fig. 2sa–c). Instead, after the 69th case, no difference in the CCI or hemoglobin level was detected. Comparison of the early (1–27) and the late phase (28–121) of the CUSUM curve for AL identified a significantly diminished rate of AL in the late phase (25.8% vs 5%, *p* = 0.005). The risk of clinical anastomotic failure was not significantly different between the first and second phases (11.5 vs 5.1%, *p* = 0.251). A second analysis tested the 47th case as a cutoff point, but the risk of AL and clinical anastomotic failure remained stable in the second phase compared to the previous analysis (17% vs 5%, *p* = 0.056 and 8.9% vs 5%, *p* = 0.458, respectively).

### Short- and long-term oncological results

After TaTME, the pathological analysis showed a rate of distal margin involvement of 0% and a positive CRM rate of 3.3%. The quality of the specimen was judged as complete/near complete in 97.5% of cases. Excluding the two postoperative deaths, among 59 patients with a minimum follow-up of 24 months for survivors, the rate of recurrence was 18.6% (median DFS = 34 months), the rate of local recurrence was 1.7%, the rate of distant recurrence was 18.6%, the rate of cancer-related mortality was 5.1%, and the rate of all-cause mortality was 11.8% (median OS = 35.1 months). None of the patients with local relapse presented a multifocal pattern. CUSUM and RA-CUSUM curves for pathological and long-term results were not plotted because there were too few events.

## Discussion

TaTME was introduced a decade ago as a surgical option for the treatment of rectal cancer to overcome some technical challenges in mesorectal dissection, particularly when surgery is carried out through a minimally invasive approach for mid/low tumors in male or obese patients [7]. From the beginning of its introduction in surgical practice, TaTME has been claimed to be “the solution for old problems” and able to serve as a new dissection technique seemingly “much easier” than the transabdominal approach, performed open or laparoscopically [26]. Moreover, the rapid success of the TaTME procedure has been boosted by the conflicting results of the last RCTs unable to establish non-inferiority of the minimally invasive approach compared to the open approach in terms of pathological outcomes [5, 6]. In the competition between open, laparoscopic, and robotic TME, TaTME has emerged as a valid alternative combining the advantages of minimally invasive surgery from the abdominal side with optimal pathological results due to better visualization of the pelvic field. As happens for every innovative procedure, TaTME has generated many concerns from a technical point of view and from an oncological perspective. Performing the pelvic phase of the surgery from below changes both the anatomical view for dissection and the anastomotic technique, possibly exposing patients to unexpected complications and increasing the risk of anastomosis. Furthermore, the distal transection of the rectal lumen inside the pelvis from the beginning of the procedure raised concerns about the oncological safety of TaTME because it may potentially expose the plane of dissection to cancer cells, no matter how tight the distal purse string [27]. The national experience of TaTME in Norway mirrored these concerns; higher than national rates of AL needing reoperation (8.4%) and local recurrence with a multifocal pattern (11.6%) led to a national moratorium on TaTME [10]. The Norway report was based on 152 patients treated at 4 centers over 4 years, meaning that approximately 10 procedures were performed per center in a year. This report raised the question of whether the recurrence rate was due to the technique itself or whether it was a learning curve effect.

A recent report from the Netherlands [11] reported the local recurrence rate during the implementation of TaTME in a structured national training program. The authors pointed out that even if the surgeons were proctored, there was a clear learning curve effect in the first ten cases, with a recurrence rate of 10% and an AL rate of 17%, which were strictly correlated with the occurrence of intraoperative complications. However, in the same study, the recurrence rate dropped to 3.8% after 40 procedures.

Excluding the Norway experience and despite the lack of high-level evidence supporting the benefits of TaTME, this technique has been widely adopted by colorectal surgeons across the world [28], and the good short- and long-term outcomes achieved at expert centers continuously support the implementation of this procedure in surgical practice.

Learning curve evaluation is one of the main areas of surgical research in TaTME, as it is such a complex surgical procedure. Identification of the learning phase of this technique is crucial because it allows recommendations for when and how to establish a training program to be made to avoid potential harm to patients and allow the supervision of surgeons not experienced in the technique.

To date, three studies focusing on the learning curve of TaTME have been published. Koedam et al. [13] reported on the learning curve in terms of the postoperative outcomes in 138 patients treated with different surgical approaches, including a single- or double-team approach and approaches involving a single surgeon in the first 80 cases and three surgeons afterward. The authors detected changes at the 40th, 100th, and 119th procedures and reported the 40th procedure as the primary cutoff, with a decrease in the MC rate from 47.5 to 17.5% and a decrease in the rate of AL from 27.5 to 5%. Instead, Lee et al. [14] focused on the quality of TaTME (including abdominoperineal resection) in 87 patients performed by 4 different surgeons using a single-team approach. They reported high-quality TME in 95% of the patients and identified a turning point for high-quality TME at the 33rd procedure and optimization after the 45th-51st procedures.

More recently, in a study of 100 patients (85 with anastomosis) treated by a single surgeon, Caycedo‐Marulanda et al. [15], reported 50 procedures as a turning point, providing a 50% improvement in the AL rate.

Overall, previous studies have reported long learning curves, pointing out the need for strict supervision in the first 40–50 cases.

This study presents a detailed learning curve analysis for TaTME. The analysis was based on a consecutive series of 121 mid-low rectal cancer patients who underwent TaTME performed by the same surgical team who adopted TaTME in April 2015 without a structured training program. Since its introduction, TaTME has been considered the first choice for treating mid-low rectal cancer. Excluding the first 8 procedures, the double-team approach with a single surgeon performing the transanal procedure was considered the standard surgical procedure for mid-low rectal cancer.

The results showed a learning curve for OT consisting of three peaks, which could be considered three predictable turning points. The first peak, at the 18th procedure, represented the end of the first phase of learning the process of a new technique. The increase could be attributed to the switch to the double-team approach and to the upgrade of the OR setup and instrumentation. The second peak, at the 54th procedure, could be considered the point at which proficiency is reached: the performance of the surgical team is optimized from a technical point of view. The third peak, at the 86th procedure, is apparently due to a case-mix effect or overconfidence in the procedure since it is consistent with the CUSUM curves for BMI, age high-risk patients undergoing TaTME during that period. Analyzing the two-sided Bernoulli CUSUM after the 71st procedure, the OT was more stable, without significant deviation from the mean. According to this trend, after the 71st procedure, the surgical team entered the “mastery” phase of the learning curve.

For MCs and reoperation, the learning curve presented three phases (1–54, 54–69, 70–121), all optimized after the 69th case. This curve was consistent with the curves for postoperative complications and readmission. For AL, the learning curve presented two phases (1–27, 28–121), with the AL rate being optimized after the 27th case.

In the first phase of the respective learning curves, a relatively higher risk of postoperative complications (46.3%), MCs (13%), and reoperation (9.3%) was detected. There were only two small alarm signals, one in the early phase for MCs and one in the early/middle phase for reoperation.

In the first phase of the learning curve (1–27), a high risk of AL (25.8%), coincident with an alarm signal, was detected. Nevertheless, most cases of AL were not clinically relevant and did not result in an equivalent risk of clinical anastomotic failure (11.5%).

Finally, the pathological and oncological results in terms of local recurrence in this case series are promising and consistent with those available from expert centers [9, 29], which provides reassurance that the technique is oncologically adequate and safe.

There are some relevant implications for the results of this study. The first is related to the observation that the initial phase of the learning curve was less safe in terms of postoperative complications and early and late AL. After the inflection point in the learning curve at the 27th case, the results for AL were acceptable and well below those reported in the literature [8, 9]. The anastomotic technique after TaTME is different from that after transabdominal rectal surgery. A radical change in the anastomotic technique is necessary after TaTME, and the results of this study reveal that this modification could be responsible for the high incidence of AL in the first phase. Fortunately, if we consider only the anastomotic complications resulting in a permanent stoma, the rate was 6.5%. Moreover, after the turning point, the AL rate significantly dropped and remained steadily low. Training programs, including tutoring and proctoring, should be implemented to mitigate the possible consequences of AL in this phase.

Second, the learning curves for the OT and postoperative outcomes were longer than that for AL, being optimized only after the 69th procedure. In the early phase, our postoperative outcomes were comparable to those in the literature, while in the middle and late phases, the rates of postoperative outcomes were below those reported in the literature, codifying a learning curve effect on postoperative management. The learning curve for these outcomes was probably longer than that of AL due to a shift in patient characteristics (i.e., more comorbidities) from the earlier to the later phases, as depicted in Supplementary Fig. 2s.

Third, the oncological outcomes of this study, albeit limited to less than half of the patients with adequate follow-up data, confirm that TaTME is a safe procedure for the treatment of rectal cancer in terms of the pathological results and recurrence rate. These results are in line with those recently published in a large multicenter study on TaTME and the local recurrence rate after TaTME, which are in net contrast with the data from the Norwegian experience [29].

The main advantage of this study is that its cohort was homogeneous with regard to the indication for TaTME, the surgical technique and the composition of the surgical team. Second, we applied a statistical methodology using different types of CUSUM control charts to avoid confounding factors and self-referentiality and to explore all the possible implications of the learning process. This study also has some limitations. First, this analyzed data represent the experience of a team composed of surgeons extensively trained in laparoscopic colorectal surgery at a high-volume center. Therefore, this curve may not be generalizable to all surgeons or institutions. Second, although this study included a reasonable number of procedures, a greater number of cases would be even more useful to identify oscillations in the surgical performance due to other causes (slight changes in the surgical technique, training of other surgeons) and to improve the quality of the O–E statistic-based regression analysis, which was limited from a few events in terms of MCs, reoperation, and AL/anastomotic failure.

## Conclusions

The learning curve for the OT was optimized after the 71st procedure. The learning curve for MCs and reoperation was optimized after the 69th procedure, while the learning curve for AL was optimized after the 27th procedure. According to our data, the first phase of the learning curve is crucial, and without adequate training, will almost certainly expose patients to a high risk of serious postoperative complications. In expert hands, the oncological outcomes of TaTME seem not to be influenced by a learning curve effect.

## Electronic supplementary material

Below is the link to the electronic supplementary material.

Supplementary file1 (TIF 1955 kb)—Figure 1s: CUSUM curves associated with reinterventions. 1sA: simple CUSUM curve, 1sB: Bernoulli Cumulative Deviation Curves for simple CUSUM, 1sC: RA-CUSUM curves, 1sD: Bernoulli Cumulative Deviation Curves for RA-CUSUM, 1sE: simple CUSUM curve using a reference mean from the literature. CumDevPosit: Cumulative sum of the positive deviations, CumDevNeg: Cumulative sum of the negative deviations, CumDevMean: mean of the CumDevPos and CumDevNeg values.

Supplementary file2 (TIF 3146 kb)—Figure 2s: CUSUM curves associated with Charlson comorbidity score (2sA), BMI (2sB), and age (2sC).
