# Supplementary figures and images for: The learning curve of TaTME for mid-low rectal cancer: a comprehensive analysis from a five-year institutional experience
Source: Surg Endosc. 2020 Oct 26;35(11):6190–200. doi: 10.1007/s00464-020-08115-0 (PMC8523384; doi:10.1007/s00464-020-08115-0)

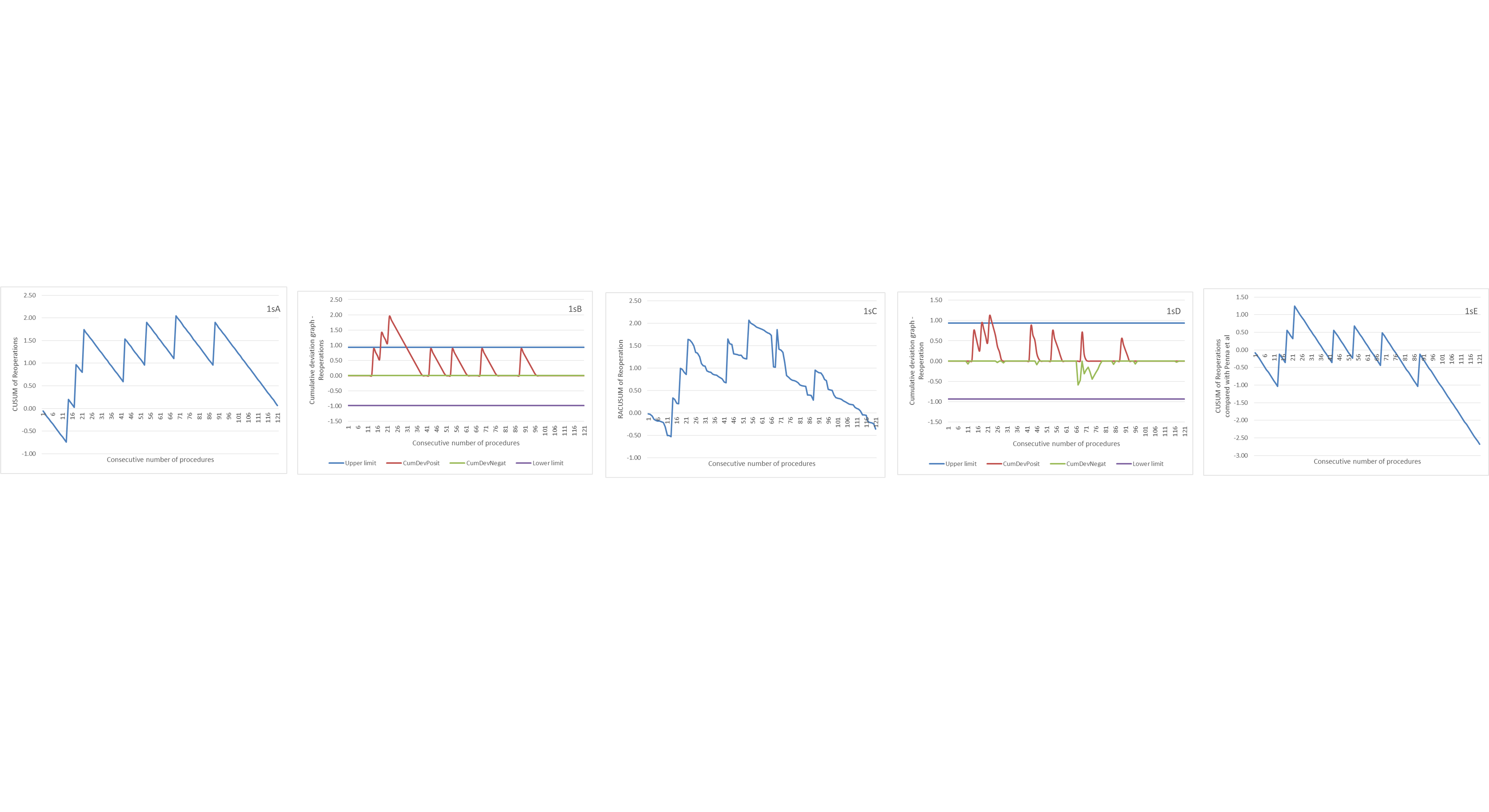

Supplement: Supplementary file 1 — Supplementary file1 (TIF 1955 kb)—Figure 1s: CUSUM curves associated with reinterventions. 1sA: simple CUSUM curve, 1sB: Bernoulli Cumulative Deviation Curves for simple CUSUM, 1sC: RA-CUSUM curves, 1sD: Bernoulli Cumulative Deviation Curves for RA-CUSUM, 1sE: simple CUSUM curve using a reference mean from the literature. CumDevPosit: Cumulative sum of the positive deviations, CumDevNeg: Cumulative sum of the negative deviations, CumDevMean: mean of the CumDevPos and CumDevNeg values. [file 464_2020_8115_MOESM1_ESM.tif]

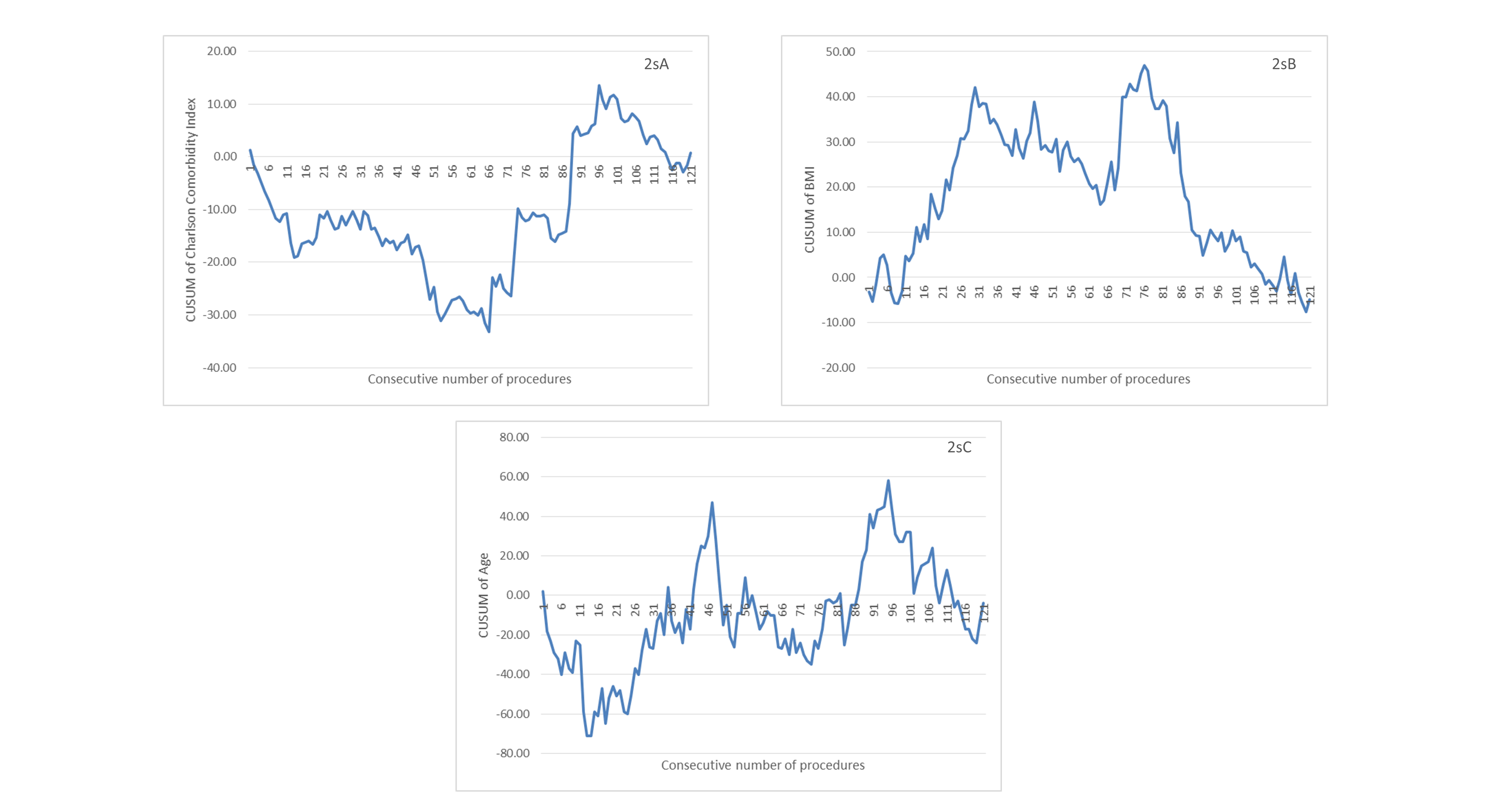

Supplement: Supplementary file 2 — Supplementary file2 (TIF 3146 kb)—Figure 2s: CUSUM curves associated with Charlson comorbidity score (2sA), BMI (2sB), and age (2sC). [file 464_2020_8115_MOESM2_ESM.tif]
